# The Journal of the American Medical Association

**Published:** 1882-08

**Authors:** 


THE JOURNAL OF THE AMERICAN MEDICAL ASSOCIATION.—During the last few years, the proposition to have the American Medical Association establish a weekly medical journal, somewhat after the form of *The British Medical Journal*, in which the proceedings and papers should be published, instead of publishing them in a single volume, as heretofore, has been urged with much earnestness by some of the most eminent members of the Association. In 1880 the subject was referred for consideration to a special committee selected for that purpose. The committee made its first report at the meeting in 1881, setting forth the probable cost of the publication of the proposed journal, the changes necessary in the by-laws of the Association, and earnestly recommending the change of publication to be made.

The report was favorably received, the committee continued and enlarged, with instructions to report a more full and definite plan of proceeding at the next annual meeting. Accordingly, at the recent meeting in St. Paul, the committee submitted its second report, still further urging the establishment of an Association Journal and the adoption of such measures as will secure its pecuniary support.

The Association adopted resolutions approving the proposed change, and appointed a board of nine trustees, with instructions to agree upon the plan for a journal, and issue a circular setting forth such plan, and address copies of the same to all members of the profession in this country, asking for direct pledges of pecuniary support. The board were also instructed to agree upon the best time and place for issuing the proposed journal, and upon the best editorial talent that could be secured for its direct management, and report definitely on all these topics at the next meeting of the association. The Board of Trustees have already agreed upon the *plan* for the proposed journal, and the circular, or prospectus, will soon be sent to members of the profession throughout the whole country. On the responses to this circular will depend the success or failure of the enterprise. If those who receive it either carelessly throw it aside without attention, or, after reading and perhaps approving it, they postpone making a reply until it is forgotten, and, in consequence, the board receive but few pledges, it cannot make a favorable report at the next meeting of the association. It is very desirable, therefore, that all members of the profession who are in favor of the publication as proposed, should make prompt replies, as requested in the circular. That a weekly journal, having a reading constituency in all parts of the country such as would be furnished by the membership of the Association, under able and judicious editorial management, would be capable of doing very much to advance all the important interests of the profession, is too apparent to need argument.

---

ONE of the editors of this journal has, during the past month made a change in the location of his office. Communications intended for Dr. James Nevins Hyde should hereafter be addressed to 240 Wabash Avenue.

---

PROF. J. ADAMS ALLEN is expected to return from Europe in time for the approaching session of Rush Medical College. Prof. H. P. Merriman of the Chicago Medical College is still abroad. Prof. W. H. Byford has returned, and is again engaged in his professional work. Prof. E. C. Dudley, of the Chicago Medical College, was on a recent date married to Miss Anna M. Titcomb, of Winnetka, Ill. He has received the warm congratulations of a large circle of friends, including his colleagues among the medical editors of this city.

